# Application of near-infrared fluorescence imaging in theranostics of gastrointestinal tumors

**DOI:** 10.1093/gastro/goad055

**Published:** 2023-09-27

**Authors:** Nan-Qing Shi, Xin-Yuan Cui, Cheng Zhou, Ning Tang, Da-Xiang Cui

**Affiliations:** Department of Sensing Science and Engineering, School of Electronic Information and Electrical Engineering, Institute of Nano Biomedicine and Engineering, Shanghai Jiao Tong University, Shanghai, P. R. China; Department of Radiology, Ruijin Hospital, Shanghai Jiao Tong University School of Medicine, Shanghai, P. R. China; Department of Sensing Science and Engineering, School of Electronic Information and Electrical Engineering, Institute of Nano Biomedicine and Engineering, Shanghai Jiao Tong University, Shanghai, P. R. China; Department of Sensing Science and Engineering, School of Electronic Information and Electrical Engineering, Institute of Nano Biomedicine and Engineering, Shanghai Jiao Tong University, Shanghai, P. R. China; Department of Sensing Science and Engineering, School of Electronic Information and Electrical Engineering, Institute of Nano Biomedicine and Engineering, Shanghai Jiao Tong University, Shanghai, P. R. China; National Engineering Center for Nanotechnology, Shanghai, P. R. China

**Keywords:** gastrointestinal cancers, early diagnosis, near-infrared fluorescence imaging, tumor margins, clinical application

## Abstract

Gastrointestinal cancers have become an important cause of cancer-related death in humans. Improving the early diagnosis rate of gastrointestinal tumors and improving the effect of surgical treatment can significantly improve the survival rate of patients. The conventional diagnostic method is high-definition white-light endoscopy, which often leads to missed diagnosis. For surgical treatment, intraoperative tumor localization and post-operative anastomotic state evaluation play important roles in the effect of surgical treatment. As a new imaging method, near-infrared fluorescence imaging (NIRFI) has its unique advantages in the diagnosis and auxiliary surgical treatment of gastrointestinal tumors due to its high sensitivity and the ability to image deep tissues. In this review, we focus on the latest advances of NIRFI technology applied in early diagnosis of gastrointestinal tumors, identification of tumor margins, identification of lymph nodes, and assessment of anastomotic leakage. In addition, we summarize the advances of NIRFI systems such as macro imaging and micro imaging systems, and also clearly describe the application process of NIRFI from system to clinical application, and look into the prospect of NIRFI applied in the theranostics of gastrointestinal tumors.

## Introduction

Gastrointestinal cancers, including esophageal cancer, gastric cancer, and colorectal cancer (CRC), have the characteristics of high morbidity and high mortality. According to the latest research statistics in 2022, esophageal cancer, gastric cancer, and CRC rank second, third, and sixth, respectively, in the incidence of cancer in China, and rank fourth, third, and fifth, respectively, in the mortality of cancer in China [1].

Under the current routine diagnostic methods, gastrointestinal cancer is often diagnosed at an advanced stage and the prognosis of advanced cancer is poor [2, 3]. Early diagnosis is an effective method to improve the prognosis of gastrointestinal cancers [4–8]. White-light endoscopy (WLE) is the most commonly used tool for detecting gastrointestinal cancers at an early stage. However, due to the limitation of white light in penetration and targeting, subtle lesions (e.g. flat adenomas) and dysplasia within fields of transformed mucosa (e.g. Barrett’s esophagus) are difficult to be clearly identified [9]. Due to inexperience and flaws in WLE, doctors occasionally make incorrect assessments of disease conditions [10]. These challenges provide the incentive for the development of new endoscope systems to make up for the shortcomings of WLE.

The principle of near-infrared fluorescence imaging (NIRFI) is that some dye molecules can be excited by light within the ultraviolet to the infrared spectrum to produce near-infrared fluorescence, which can be received by special optical sensors [11]. Compared with white light, near-infrared fluorescence can visualize deep-tissue structures because of its stronger tissue penetration [12]. Compared with traditional fluorescence imaging, NIRFI can avoid the biological autofluorescence background, which is helpful to improve the signal-to-noise ratio and the sensitivity of imaging [13]. Aiming at the lack of tumor specificity of fluorescent dyes, in the recent research of NIRFI, researchers combined fluorescent dyes with target molecules to develop molecular targeted fluorescent tracers [14]. Therefore, NIRFI technology can make up for the deficiency of WLE in the diagnosis of minimal lesions and is of great significance for improving the early diagnosis rate of gastrointestinal tumors.

In addition to gastrointestinal tumor diagnosis, NIRFI also has many applications in gastrointestinal surgery. Surgical resection is one of the important treatment methods for gastrointestinal tumors. The recognition of tumor location and lymph node (LN) metastasis, as well as the confirmation of anastomotic conditions, has a great impact on the therapeutic effect of surgery. At present, the treatment of these surgical conditions mainly depends on the subjective judgment of doctors, which might result in post-operative recurrence and metastasis. In addition to the molecular targeted fluorescent tracers mentioned above, near-infrared fluorescent molecules can also be imaged in tumors according to an enhanced permanency and retention (EPR) effect [15]. With the help of EPR, tumors can be accurately identified. Based on this, NIRFI has great application prospects in gastrointestinal surgery.

To date, some reviews have discussed the application of molecular imaging in the gastrointestinal tract [16–22] and some have discussed the application of fluorescence-guided surgery [23–29]. Few reviews have focused on the application of NIRFI in the diagnosis and treatment of gastrointestinal tumors.

Herein, we review the latest progress in near-infrared fluorescence endoscopic imaging systems for the diagnosis and surgical treatment of gastrointestinal tumors; review the main advances in the application of NIRFI in the early diagnosis and surgical navigation of esophageal cancer, gastric cancer, and CRC; and discuss the application prospects, the concepts, issues, approaches, and challenges, with the aim of improving to develop NIRFI technology for precise diagnosis and therapy of gastrointestinal tumors.

## Near-infrared fluorescence endoscope systems

At present, endoscope systems used for NIRFI are mainly divided into two categories: macro and micro NIRFI systems [30, 31]. With the help of macroscopic imaging, doctors can quickly scan the whole organ in a wide field of vision and identify suspicious lesions. Microscopic imaging can further identify suspicious lesions at the cellular scale. In order to integrate the advantages of various imaging methods, the macro NIRFI system often adopts multimodal imaging modes [32–42].

**Figure 1.**
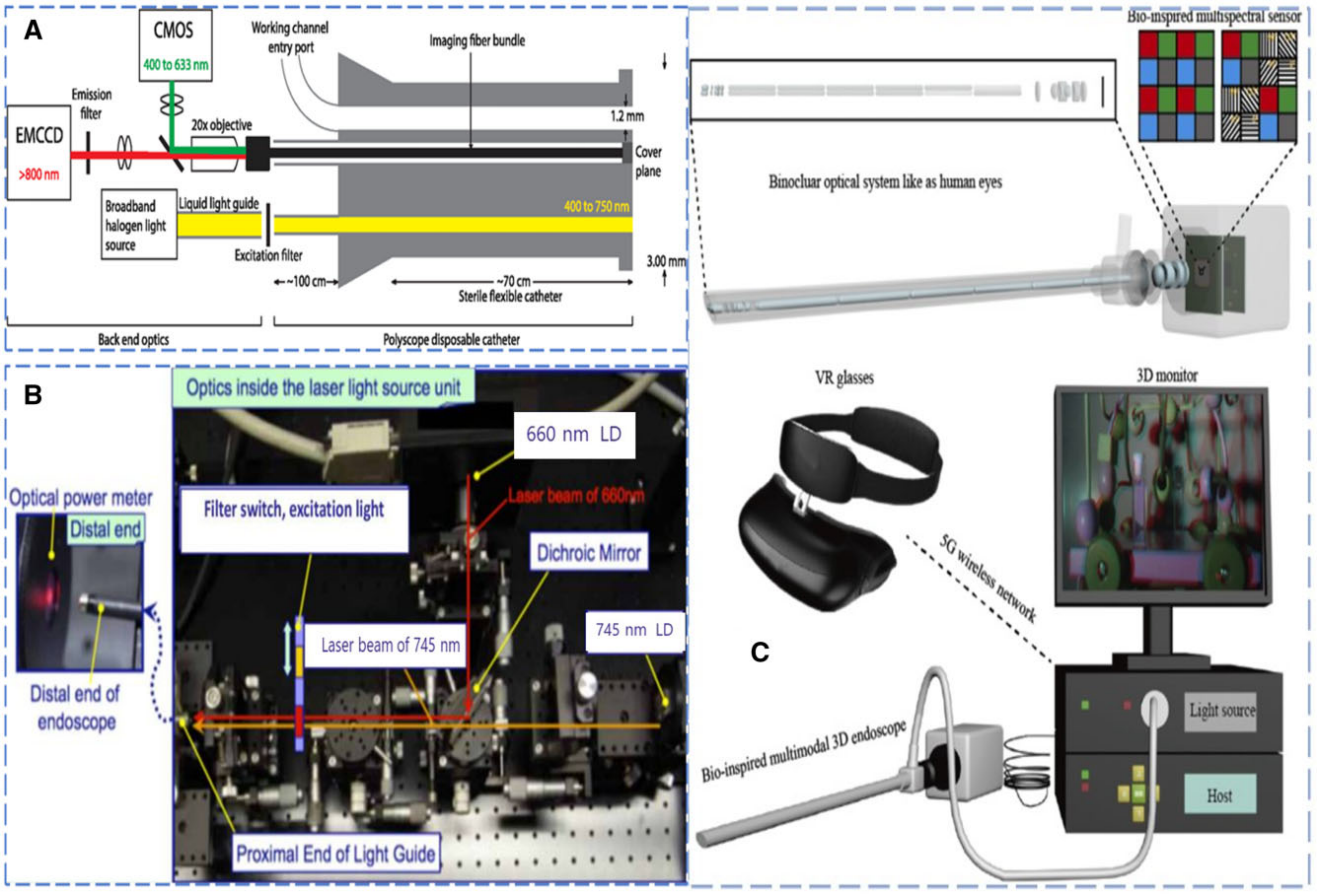
Endoscope systems. (A) Hardware diagram of a bimodal endoscopic imaging system. (Reproduced from Waterhouse *et al*. [40], 2016 with the permission from SPIE.) (B) Connection diagram of 745-nm laser and 660-nm laser. (Reproduced from Shrivastav *et al*. [41], 2018 with the permission from PLOS.) (C) Diagrams of the 3D endoscopic imaging system macro and the 3D image created by combining color and near-infrared images. (Reproduced from Liu *et al*. [42], 2021 with permission from Opt Express.).

To overcome the contrast defects of the white-light endoscope and the autofluorescence endoscope, Waterhouse and colleagues [40] developed a convertible dual-mode endoscope system with a field of view of 63 degrees and an image resolution of 62 μm (Figure 1A). The system uses a diffusion series of wheat germ agglutinin (WGA-IR800cw) to ensure a minimum detectable concentration of 110 nmol/L. Through the imaging experiment in the isolated mouse stomach, it has been proven that the system can well distinguish the normal gastric mucosa and squamous tissue, and a small-scale experiment in patients with Barrett esophageal cancer proved that the system could distinguish the disease pathology in esophageal tissue [40].

In the NIRFI system, the selection of fluorescence probes has a great impact on the imaging effect. In order to determine the efficiency of various near-infrared fluorescence probes in the same lesion, Shrivastav and colleagues [41] developed a double-laser near-infrared fluorescence endoscope. The system has two light sources of 660 and 747 nm (Figure 1B) and two band-pass filters of 400–680 and 720–750 nm form an excitation filtering system, which realizes two inspection modes with wavelengths of 680 and 750 nm, and the imaging frame rate reaches 10 frames per second. The experimental results confirm that the double-laser near-infrared fluorescence endoscope is a powerful probe verification tool, which has great application potential in selecting probes for detecting abnormal lesions in early tumors [41].

In addition to being used for cancer detection, the macro NIRFI system also has a strong application prospect in clinical surgery guidance. In order to solve the coexistence of normal surgical lighting and NIRFI, Liu and colleagues [42] proposed a bionic multimodal 3D imaging system. The system combines the advantages of human eyes and the compound eyes of the mantis shrimp. A broad-band binocular optical system, an optical relay system, and a bio-inspired multiband sensor constitute the core of the system (Figure 1C). The system uses white light with a wavelength of 400–700 nm and a near-infrared laser with a wavelength of 785 nm as the light source at the same time. The white light realizes RGB (red, green, blue) images with high contrast. The near-infrared light source is combined with a notch filter to obtain the near-infrared fluorescence image. The two pairs of images are fused together and then the 3D image is obtained through the 3D algorithm. The experimental results confirm that the 3D endoscope system can realize real-time imaging of tumor tissue and LN location [42].

The commonly used microscopic NIRFI system is confocal laser endomicroscopy (CLE) [43–46]. CLE uses a low-power laser as the light source, and the laser irradiates the tissue and then reflects it back to the equipment. The core principle of CLE is confocal. The light source and the reflected light collection system will be on the same focal plane and only the reflected light refocused through the same lens will be detected by the device. This way greatly improves the spatial resolution of CLE, enabling CLE to achieve optical biology and *in vivo* history [46].

For example, to break through the limitation of imaging depth and realize deep-tissue imaging at the cellular level for gastrointestinal tissue, Wang and colleagues combined the near-infrared probe with CLE and developed a set of NIRFI system [47]. The system includes a 785-nm laser, a 2D scanning system composed of two scanners, and a band-pass filter. The imaging speed can reach five frames per second and the resolution is 1,024 × 1,024 pixels [47]. Due to the weak scattering effect of near-infrared light in deep-tissue imaging, the system can be applied to image esophageal mucosal muscularis under a submucous depth of 250 μm, and can also be used to identify normal tissues in colon and abnormal tissues in ulcerative colitis under a submucous depth of 300 μm [47]. The system shows great potential in the real-time diagnosis of digestive tract diseases.

## Early diagnosis

### Esophageal cancer

Esophageal adenocarcinoma (EAC) is one of the most common malignant tumors of the digestive tract, with a 5-year survival rate of <15% [48]. Early detection of pathological changes in EAC has an important impact on improving prognosis. Barrett esophagus (BE) is the only known precursor of EAC. The risk of EAC in patients with BE is >30 times that in ordinary people. Therefore, it is very important to screen patients with BE in the population [49]. At present, WLE diagnosis is mainly carried out for BE. WLE diagnosis requires regular detection and it has encountered certain challenges in accurate identification [21]. Based on this, it is of great significance for the accurate and specific recognition of BE. Recently, there have been many studies on the application of NIRFI in BE detection [50–57].

**Figure 2.**
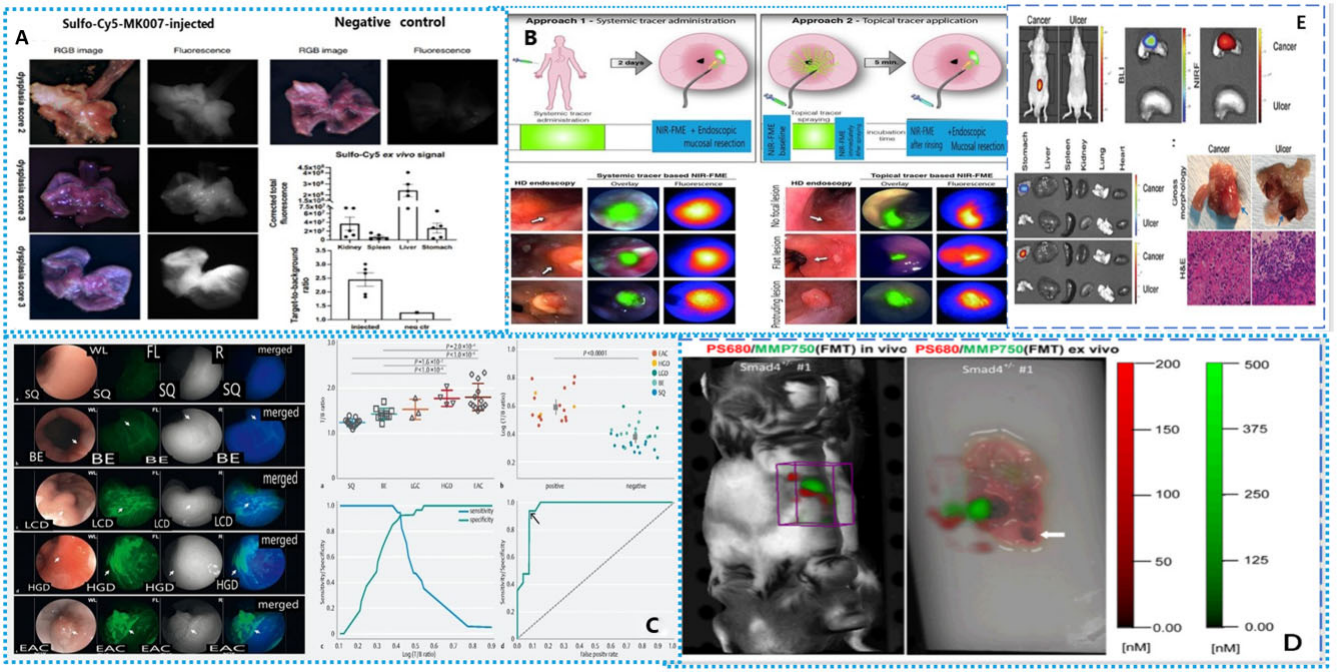
Early diagnosis in esophageal cancer. (A) Fluorescence imaging diagram and TBR results of three mice. (Reproduced from Marcazzan *et al*. [55], 2019 with permission from Gut.) (B) Schematic diagram and imaging effect diagram of two imaging methods. (Reproduced from Nagengast *et al*. [56], 2017 with permission from Nagengast.) (C) Imaging comparison of gastric cancer and gastric ulcer in mice and fluorescence intensity analysis of various organs. (Reproduced from Chen *et al*. [57], 2022 with permission from Thieme.) (D) Imaging examples and mathematical analysis of five pathological patterns of esophageal cancer. (Reproduced from Ding *et al*. [62], 2012 with permission from SAGE.) (E) Schematic diagram of fluorescence imaging *in vivo* and *in vitro* of mice and comparison of fluorescence intensity. (Reproduced from Zhao *et al*. [63], 2016 with permission from Molecular Imaging.).

In order to overcome the shortcomings of WLE and random biopsy in early EAC detection, Marcazzan and colleagues [55] conducted near-infrared fluorescence endoscopy diagnosis in mouse models. Chemokine receptor 4 (CXCR4) was selected as the receptor in the experiment. CXCR4 was overexpressed in EAC and showed the pathological progression from BE to EAC. The near-infrared dye sulfo-cy5 was selected as the contrast agent. Sulfo-cy5 performs extremely well in brightness and has stronger tissue permeability than traditional near-infrared dyes similar to indocyanine green (ICG). Color and NIRFI and confocal microscope fluorescence imaging were performed in mice, respectively (Figure 2A). The experimental results showed that the fluorescence signal was mainly accumulated in the cells near the dysplastic lesions. The experimental data confirmed that CXCR4 targeted near red fluorescence imaging had the ability to identify dysplastic lesions in patients with BE.

Conventional high-definition white-light endoscopy (HD-WLE) can only recognize the visualization of surface morphological changes, whereas current fluorescent molecular endoscopy (FME) can realize cancer-specific pathological subsurface examination. Based on this, Nagengast and colleagues [56] proposed a NIRFI combined with FME to improve the detection of early esophageal lesions. The test used vascular endothelial growth factor A (VEGFA) as the target of BE and used near-infrared–fluorescent 800CW as the fluorescent dye (Figure 2B). The results showed that this NIRFI-FME method improved early lesion detection by 33% compared with the traditional HD-WLE, showing the potential of detection on flat and difficult-to-distinguish lesions [56].

There are great differences in the expression level of a single target between different patients, even between the same patient. Simultaneous imaging of multiple targets shows potential in improving diagnostic performance. Chen and colleagues [57] proposed a near-infrared fluorescent peptide for double-target NIRFI. The system integrates two unique ligands such as epidermal growth factor receptor (EGFR) and ErbB2 into the same entity, and only one imaging agent is required for imaging. The difference in the emission spectrum between IRDye800cw and standard white light enables doctors to observe the target at the level of white light and fluorescence at the same time. At a target-to-background ratio of 1.5, the sensitivity and specificity of BE were 94.1% and 92.6%, respectively (Figure 2C). Experiments showed the feasibility of this dual target imaging method in early BE detection.

### Gastric cancer

Gastric cancer was the sixth most common cancer and the third leading cause of cancer death worldwide in 2022 [58]. Although the incidence of gastric cancer has declined in the past few decades, the 5-year survival rate of gastric cancer is still very low. In developed countries such as Japan, the early diagnosis rate of gastric cancer can reach 50% and the 5-year survival rate can reach 90% [59]. Early screening of gastric cancer is of great significance. Several gastric cancer studies have demonstrated the potential of NIRFI in the early screening of gastric cancer [60–63].

To develop a highly sensitive and specific NIRFI system for early gastric cancer screening, Ding and colleagues [62] validated the feasibility of NIRFI for gastric cancer screening using Cathepsin-activatable and matrix metalloproteinase (MMP)-activatable molecular probes in a mouse model. In the experiment, three Smad^+/–^ mice were injected using a Prosense680 probe and an MMP Sense 750 probe, respectively, and then *in vivo* and *in vitro* NIRFI were performed, respectively (Figure 2D). According to the experimental results in the control mice, the MMP probe can be activated by gastric dysplasia and adenocarcinoma, and the cathepsin probe can be activated by hyperplastic and dysplastic lesions and adenocarcinoma, demonstrating the feasibility of improving clinical gastric tumor detection [62].

To address the issue of distinguishing gastric cancer from gastritis in clinical diagnosis, Zhao and colleagues [63] developed a NIRFI heptamethine carbocyanine dye (MHI-148). In *in vivo* imaging of gastric ulcer mice and gastric cancer mice, strong NIRFI signals were clearly captured from gastric tumors, whereas no fluorescence was detected in gastric ulcers (Figure 2E). The gastric ulcer signal intensity decreased 6-fold relative to gastric cancer *in vitro*. At the same time, it did not show specificity in other organs of mice such as the liver, spleen, kidneys, lungs, and heart [63]. These results demonstrate the potentially unique use of NIRFI dyes for gastric tumor detection.

### CRC

CRC is one of the main causes of cancer-related death. General investigation showed that most CRC changes slowly from pre-body lesions [22]. However, the missed diagnosis rate of routine endoscopic screening of CRC and physical examination before CRC is as high as 30% [64]. NIRFI is an effective detection technology for CRC and its precursors [64–72].

In order to specifically detect other polyps missed in the detection, Burggraaf and colleagues [70] designed a water-soluble probe GE-137 composed of 26 amino acid cyclic peptides, which can specifically aggregate in tumors with c-met expression. A pilot study was carried out in 15 patients with colorectal tumors. A total of 98 lesions were found; 100% (47/47) adenomas and 78% (33/42) enhanced polyps showed obvious fluorescence enhancement, and nine adenomas were only visible in near-infrared fluorescence. The experiment confirmed that NIRFI based on GE-137 was a suitable means for the molecular detection of colorectal tumors [70].

For polypoid and flat precancerous lesions, Rabinsky and colleagues [71] studied the overexpression of Claudin-1 in precancerous lesions of colon cancer and carried out near-infrared real-time imaging verification. Cy5.5 was used as the dye in the experiment, combined with peptide molecule RTSPSSR for targeted imaging. *In vivo* experiments in five mice showed that the fluorescence intensity from polyps increased significantly and *in vitro* experiments in three mice showed that the target-to-background ratio of dysplastic sites increased significantly. Fluorescence detection in human colon samples confirmed that the average fluorescence intensity of adenomas and proliferative polyps was higher [71].

Near-infrared fluorescence greatly enhanced the contrast between normal mucosa and dysplastic mucosa. Tjalma and colleagues [72] used vascular endothelial growth factor A (VEGF-A) and EGFR as targets and irdye800cw as a fluorescent agent for the detection of colonic polyps. The experimental results showed that these two targets were overexpressed in colorectal lesions. The imaging results on the NIRFI endoscopic platform showed that the tumors in the control group could be specifically recognized. The results of preclinical experiments confirmed the feasibility of these two targets for visualization of small colon tumors [72].

## Gastrointestinal surgery

### Tumors margin recognition

Surgery is the main treatment for most gastrointestinal tumors [73]. In order to completely remove the tumor site, the surgeon must accurately identify the tumor boundary. For example, the overall 5-year survival rate of esophageal cancer is <20%; the survival rate after surgical resection can reach 50%, whereas resection with a positive resection edge often leads to a lower survival rate [74]. At present, there are a lot of studies on the application of NIRFI in image-guided surgery.

**Figure 3.**
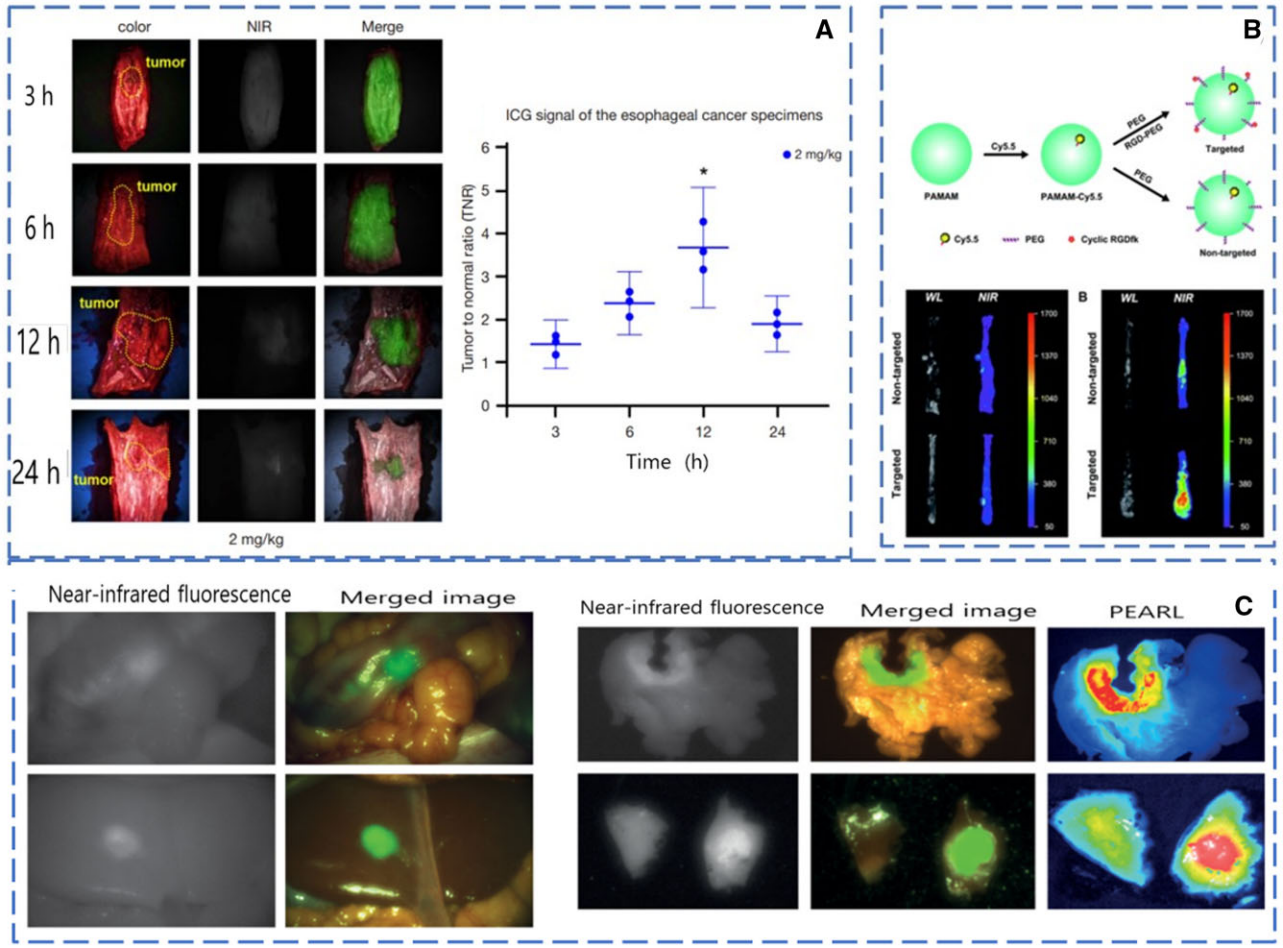
Tumor margin recognition in surgery. (A) Fluorescence diagram and fluorescence intensity analysis of fresh resected esophageal cancer specimens. (Reproduced from Rho *et al*. [75], 2021 with permission from Rho.) (B) Schematic diagram and fluorescence intensity diagram of Cy5.5 target combination with cancer cells. (Reproduced from Li *et al*. [76], 2016 with permission from RSC.) (C) Schematic diagram of tumor margin fluorescence imaging, merged imaging, and pearl imaging. (Reproduced from Boogerd *et al*. [77], 2018 with permission from Elsevier.).

In order to evaluate the ability of ICG in NIRFI during esophageal cancer surgery, Rho and colleagues [75] conducted imaging experiments in rabbits and patients with esophageal cancer. In the experiment, 45 rabbits and 12 esophageal cancer volunteers were tested for fluorescence intensity. Fluorescence signals were detected in all samples. The results of tumor-to-normal ratio (TNR) analysis showed that the dose of ICG was positively correlated with the TNR (Figure 3A) and no serious complications occurred in any patients. The experimental results showed the feasibility of detecting esophageal tumors by using intravenous ICG [75].

ICG can also be used for intraoperative tumor recognition in colon cancer surgery. Watanabe and colleagues [40] performed ICG-guided tumor resection on 80 patients with CRC. In the high-definition video laparoscope system, NIR fluorescence images can be displayed in white-light view in pseudogreen color and the fluorescence region does not diffuse. Statistical results showed that ICG fluorescence imaging can accurately identify the tumor location of colon cancer.

However, relatively rapid clearance of ICG is the main disadvantage of ICG. A targeting probe combined with a fluorescent agent is a new method for intraoperative tumor identification.

Li and colleagues [76] developed a targeted fluorescent probe for esophageal squamous cell carcinoma (ESCC) by combining cy5.5 with cyclic RGDfK peptide. The experiment in the mouse esophageal tumor model confirmed that, compared with white-light imaging, NIRFI increased the tumor-to-background ratio (TBR) for *in vivo* imaging from 1.69 to 5.09, and the fluorescence intensity of the targeted probes was 2.27 times that of non-targeted probes, which showed that the targeted probe had obvious advantages in recognizing the surgical edge of the esophageal tumor compared with non-targeted probes (Figure 3B).

Boogerd and colleagues [77] made a near-infrared fluorescence targeting probe SGM-101 according to the overexpression of carcinoembryonic antigen (CEA) in CRC. Fluorescence imaging experiments were carried out in 17 patients. Among 43 lesions, 19 were detected by using fluorescence imaging. The average TBR was 6.10, sensitivity was 98%, specificity was 62%, and accuracy of the fluorescence intensity was 84% in the expansion cohort. SGM-101 can improve the results of radical resection of CRC (Figure 3C) [77].

In addition to targeted probes, near-infrared fluorescent clip (NIRC) is also a strategy for intraoperative tumor localization. For example, in order to solve the deficiency of intraoperative endoscopy in gastric tumor localization, Narihiro and colleagues [78] used NIRC to mark the tumor site during gastrectomy and conducted clinical experiments. In an 81-year-old female patient with early gastric cancer, NIRC was used for gastrectomy. Under the guidance of NIRC, doctors successfully identified the proximal and distal cutting edges, and were able to remove each sample individually. Resection was also successfully performed in an 81-year-old male patient. There were no adverse reactions in patients after operation and the treatment effect was good [78].

### LN detection

The sentinel lymph node (SLN) is the first drainage station for primary tumor metastasis [79]. An experiment in 223 melanoma patients in 1992 introduced the concept of SLN and then, in 107 breast cancer patients, SLN surgery was also performed; SLN biopsy has now begun to be extended to gastrointestinal cancer [80, 81]. Existing imaging methods can identify large LNs, but are insufficient in accurately identifying LN metastases. NIR-fluorescent imaging offers a problem-solving feasibility.

**Figure 4.**
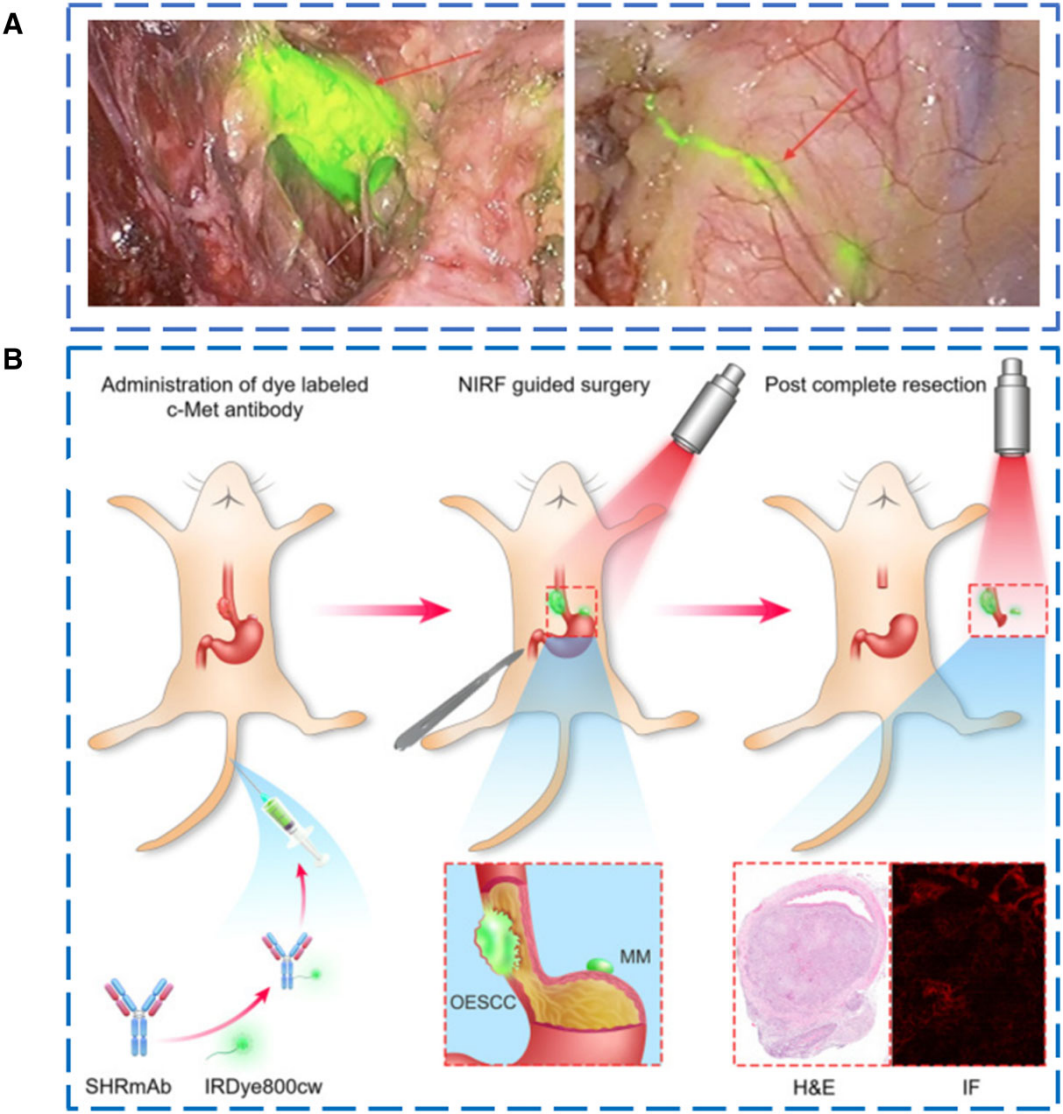
Lymph node detection in surgery. (A) Schematic diagram of fluorescence imaging of lymph nodes *in vivo*. (Reproduced from Jiang *et al*. [82], 2020 with permission from Springer Nature.) (B) Schematic diagram of lymph node imaging during surgery. (Reproduced from Liang *et al*. [83], 2021 with permission from Elsevier.).

To evaluate the feasibility of NIRFI to detect SLNs during ESCC surgery, Jiang and colleagues [82] used ICG to perform LN imaging experiments in 21 patients with confirmed ESCC and NIR^+^ LN was detected in 20 of them, confirming the efficacy of NIR in lymphatic mapping (Figure 4A) [82].

ICG does not specifically recognize metastatic LNs. In order to accurately identify LN metastasis in ESCC, Liang and colleagues [83] synthesized a targeting probe SHRmAb-IR800 conjugated with a NIR dye and c-Met antibody for NIR-guided ESCC surgery and performed surgical experiments in a mouse model (Figure 4B). The sensitivity and specificity of NIRFI-guided resection of metastasis derived from orthotopic cancers were 92.00% (46/50) and 89.74% (35/39), respectively. These results suggest that NIR imaging targeting c-Met protein can be used to differentiate benign LNs from metastatic LNs in ESCC patients [83].

ICG also has a considerable number of applications in the detection of gastric cancer SLNs [84–88]. Jung and colleagues [84] conducted an evaluation of the diagnostic value of ICG-guided LN detection in gastric cancer. Data were collected from 592 patients between 2013 and 2016, all of whom underwent endoscopic surgery before surgery and ICG injection followed by LN resection under fluorescence imaging. Among them, 150 patients developed LN metastasis; NIR angiography was achieved in 95.3% (143/150) of these patients and the negative predictive value of non-fluorescent LNs reached 92%. The experimental results demonstrated the promise of ICG-guided NIR-LN imaging in LN resection [84]. A series of achievements in the clinical application of gastric cancer lymphatic detection have been reported [89–91]. ICG was used as a tracer for NIRFI of LNs to guide laparoscopic lymphadenectomy. In a controlled experiment on 514 patients, the results showed that NIRFI significantly improved the detection rate of LNs [89]. The results of another controlled clinical trial in 266 gastric cancer patients showed that ICG did not increase complications after clinical LN resection and the safety of NIRFI was verified [90].

SLN detection in CRC surgery is also of great significance and the application of NIR imaging technology has promoted the development of SLN detection technology in colorectal surgery. For example, Currie and colleagues [92] performed NIR-SLN imaging in colon cancer surgery using ICG as a fluorescent agent. Experiments were performed in patients with stage T1 and T2 colon cancers with ICG injections around the tumor before surgery. Thirty patients were examined, of whom 90% (27/30) completed SLN mapping, confirming the efficient detection of colonic SLN by using NIR imaging. In addition to subcutaneous injection, intravenous ICG can also be used for SLN detection in colon cancer. For example, Cao and colleagues performed preoperative ICG injection in 11 patients and obtained a total of 40 SLNs, with 95% (38/40) of LNs being detected [93]. These results also showed the feasibility of NIR-fluorescent imaging in the detection of SLNs in colon cancer.

### Prevention of anastomotic leakage

Anastomotic leakage (AL) is one of the most worrisome complications of gastrointestinal surgery; because of its high morbidity and mortality characteristics, AL places enormous pressure on both patients and physicians [94]. In esophagectomy, the ischemic condition of the conduit is an important factor in the development of AL; NIRFI can visualize blood flow intraoperatively to assist physicians in surgery to prevent AL [95]. In CRC surgery, better observation of vascularization of the anastomosis will help prevent anastomosis leakage and fluorescent angiography given to ICG during surgery appears to improve the outcome of laparoscopic anastomotic surgery in terms of safety and efficiency [96]. At present, surgeons have not found effective means to predict AL in gastrointestinal surgery. A study by Karliczek and colleagues [97] showed that, when allowing surgeons to predict AL in 191 patients undergoing CRC surgery, the prior rate was only a 14% risk. Therefore, we urgently need better AL testing methods. The use of NIR fluorescence imaging to guide gastrointestinal surgery can effectively reduce the risk of AL [97].

The incidence of AL in esophagectomy ranges from 5% to 20% [98] and NIR-guided angiography has a certain effect in predicting AL after esophagectomy. For example, Koyanagi and colleagues [99] used ICG to perform angiography in 40 patients to predict the development of AL by assessing the flow speed of ICG fluorescence in the gastric conduit and found that, among 18 patients, 15 developed flow slowing and AL occurred in 7 of them (*P *<* *0.001). These results suggest that NIR imaging may be used to predict AL after esophagectomy.

NIR angiography is more widely used in reducing the incidence of AL after colorectal surgery [100–103]. To reduce the risk of AL after colorectal surgery, Impellizzeri and colleagues [102] tested a cohort of 196 CRC surgery patients, 98 of whom did not use NIR-ICG, with a physician's visual inspection to determine the location of resection. Ninety-eight patients underwent intravenous ICG and NIR imaging, followed by physician judgment for anastomotic perfusion. AL occurred in 6% (6/98) of patients who did not undergo NIR-ICG, whereas none of the patients who underwent NIR-ICG developed AL. This study demonstrated that the use of NIR angiography significantly reduces post-operative AL rates [102]. How to quantitatively evaluate anastomotic perfusion in CRC surgery by using NIR-ICG angiography is an important issue. Hayami and colleagues [103] performed NIR angiography in 69 patients and evaluated the following data: maximum fluorescence (Fmax), time from ICG injection to Fmax (Tmax), and time from start of dyeing to Fmax (ΔT). In addition, a vessel staining pattern classification system (VSPCS) was established to perform imaging at four different contrasts. The experimental results showed that there was no significant correlation between Fmax, Tmax, ΔT and AL, and the VSPCS system had a quantitative evaluation effect on anastomotic perfusion [103].

Recently, have also been some studies on NIR angiography for the evaluation of AL in gastric cancer surgery [104–106]. For example, to address the quantitative assessment of AL by using NIR-ICG imaging in gastric cancer surgery, Spota and colleagues [106] studied 100 patients undergoing gastric cancer surgery. Three variables were determined: the first time point (FT) at which the first NIR fluorescence appeared, the second time point (ST) at which the second NIR fluorescence appeared, and the time difference between FT and ST, which was defined as TD. Three NIR imaging modes are defined: homogeneous pattern, heterogeneous pattern, and fatal pattern. These elements were subjected to univariate and multivariate analyses. The results of univariate analysis showed that AL had nothing to do with ST, the three imaging modalities had significant differences in predicting AL (*P *=* *0.001), and TD had excellent performance in both univariate and multivariate analysis and could be used to predict AL in gastric cancer surgery. The experimental results demonstrate the feasibility of TD in predicting AL in gastric cancer surgery [106].

## Clinical application and new methods

### Clinical application

ICG is currently the most common fluorescent agent in NIRFI, but its specificity needs to be improved [105]. NIRFI with the use of tumor-targeted fluorescent agents can play an essential role. At present, there are already some new fluorescent molecular targeted probes for NIRFI under development.

In order to achieve clinical translation, the following work needs to be done well. The toxicological profile is required for any first-in-human investigation in order to establish a safe starting dose for humans. Most preclinical investigations recognize the maximum tolerated dose, particularly for new drugs. The maximal tolerable dose is not always necessary for fluorescent agents to produce the best results, which is a significant distinction between fluorescent agents and therapeutic research medications. For the medicine to have the desired effect, the proper dosage must be determined. The clinical transformation of innovative selective fluorescence probes depends on achieving a high target-to-surrounding-tissue ratio with modest dosages of fluorescent chemicals.

Additionally, contemporary imaging systems are needed for the clinical translational procedure. Even though NIRFI imaging system development has made significant strides, the software and hardware of the system still need to be tuned to make it more portable. To maximize tumor detection during surgical procedures, laparoscopic or endoscopic equipment must incorporate imaging technologies that adhere to the trend toward minimally invasive surgery.

### New methods

Although NIRFI allows precise tumor imaging, doctors' attention spans may greatly affect detection rates due to endoscopists' limitations as individuals [107]. Therefore, the combination of NIRFI and artificial intelligence (AI) detection is an important direction for the future. In addition, AI can also be used to dynamically perfuse exogenous substances to reveal tissue-specific patterns, overcoming the dependence on fluorophore clearance pharmacokinetics [108]. Therefore, the combination of AI and NIRFI is a general direction for the future.

The utilization of nanoparticles offers a novel tool for the imaging of malignancies for diagnostic purposes since materials acquire special characteristics at the nanoscale. Particles of any substance with a diameter of between 1 and 100 nm are considered nanoparticles because of their remarkable physical and chemical characteristics. Due to their tiny size, adaptable surface features, and retention effects, nanoparticles such as quantum dots, iron oxide nanoparticles, and gold nanoparticles have recently been very useful in gastrointestinal cancer imaging. Studies using nanoparticles in conjunction with conventional imaging techniques for gastrointestinal cancers have greatly increased the rate of early diagnosis and staging accuracy [109].

Especially as nanotechnology develops very fast, some nanoprobes combined with NIRFI and photothermal therapy or photodynamic therapy have been designed and developed for imaging directed therapy of gastrointestinal tumors and have clinical translation prospects [110–112]. Therefore, the combination of nanotechnology and NIRFI is also a new direction for the future.

As shown in Table 1, near-infrared fluorescence endoscopic imaging systems used for imaging diagnosis of gastrointestinal cancer are summarized. In order to realize the precise diagnosis and treatment of gastrointestinal tumors, a new generation of intelligent NIR-fluorescent endoscopic imaging systems combined with targeted nanoprobes and a wireless energy supply should be the development direction for the future [113, 114].

**Table 1.** Summary of near-infrared fluorescence endoscopic imaging system of gastrointestinal cancer

Reference	Technique	Study	Imaging agent	Year	Brief description
Fengler [32]	White-light and fluorescence imaging	Colorectal cancer	ICG	2015	High-definition video laparoscopy system, designed by Fengler in Canada
Glatz *et al.* [33]	White-light and fluorescence imaging	Colorectal cancer	ICG	2013	Concurrent video-rate color and near-infrared fluorescence laparoscopy, designed by Juergen in Germany
Gounaris *et al.* [34]	White-light and fluorescence imaging	Colonic dysplasia	Cy5.5	2016	Near-infrared fluorescence endoscopy, designed by Gounaris in USA
Li *et al.* [36]	Optical coherence tomography and fluorescence imging	Colorectal cancer	ICG	2019	Optical coherence tomography and near-infrared multimodal endoscopy, designed by Li in USA
Liu *et al.* [42]	3D stereoscopic, white-light, and fluorescence imaging	Image-guided and robotic surgery	ICG	2021	Bio-inspired multimodal 3D endoscope, designed by Liu in China
Shrivastav *et al.* [41]	Dual laser fluorescence imaging	Colon cancer	Cy5.5 and Cy7.0	2018	Dual laser NIRF endoscope, designed by Shrivastav in USA
Venugopal *et al.* [38]	White-light and fluorescence imaging	Minimally invasive surgery	ICG	2013	Optimized simultaneous color and near-infrared fluorescence rigid endoscope, designed by Venugopal in USA
Waterhouse *et al.* [40]	White-light and fluorescence imaging	Barrett’s esophagus	IR800CW	2016	Near-infrared fluorescence endoscope, designed by Waterhouse in UK
Jones *et al.* [45]	White-light and fluorescence imaging	Colorectal cancer	Cy7.5	2018	Near-infrared fluorescence endoscope, designed by Jones in USA
Wang *et al.* [47]	Probe-based confocal microendoscope	Colon cancer	ICG	2018	Near-infrared probe-based confocal microendoscope, designed by Wang in China
Calcara *et al.* [113]	Blue-green-light and fluorescence imaging	Gastric cancer	ICG	2023	A total of 580 patients involved feasibility study, performed by Calcara in Italy
Huang *et al.* [114]	Near-infrared, green fluorescence, and color-segmented fluorescence imaging	Gastric cancer	ICG	2021	A total of 329 patients involved feasibility study, performed by Huang in China

ICG, indocyanine green; NIRF, near-infrared fluorescence imaging.

## Conclusion and future directions

In this review, we evaluated the most recent NIRFI developments in gastrointestinal cancer, including a near-infrared endoscopic imaging system, early tumor detection, and gastrointestinal tumor surgery. These techniques have great application prospects in improving the diagnostic rate and the effect of surgical treatment of gastrointestinal tumors. The near-infrared endoscope imaging system can recognize small lesions that the traditional white-light endoscope cannot detect and can conduct real-time navigation in surgery. Combining NIRFI with more other modes is a new tendency for the near-infrared endoscopic imaging system. By integrating the advantages of multiple imaging modes, better imaging effect can be achieved. For cancer detection, NIRFI-assisted diagnosis has been actively applied for various gastrointestinal tumors, but the development of targeted fluorescent molecular probes has limited the development of imaging technology. Future research should concentrate on developing novel probes that can be paired with tumor targets. For the application of ICG in gastrointestinal surgery, there are many cases of using ICG in the surgical guidance of esophageal cancer, gastric cancer, and CRC, which has very good application prospects. However, the research on the quantitative standard of surgical guidance is still insufficient.

In summary, NIRFI has been actively explored to apply in theranostics of gastrointestinal tumors and has made great advances; it will have better application prospects in precise theranostics of gastrointestinal tumors in the near future. We believe that NIRFI can reduce the threat of gastrointestinal tumors to human health and provide opportunities to improve the quality of human life as the rapid development of NIRFI technology is integrated with new targeted nanoprobes.

## Authors’ Contributions

N.Q.S., C.Z., and N.T. were involved in the initial drafting of the manuscript; X.C. selected the figures; and D.X.C. suggested writing this review and revised the manuscript. All authors were involved in the final approval of the manuscript.
